# Proliferative glomerulonephritis with monoclonal IgG Lambda deposits caused by plasmablastic lymphoma: a case report

**DOI:** 10.1186/s12882-023-03351-7

**Published:** 2023-10-06

**Authors:** Ling-Yan Ren, Qi Chen, Feng-Ping Qiu, Zhen-Yu Jiang, Xiao-Yi Wang, Xiao-Lan Zhang, Zhan-Qin Shi

**Affiliations:** 1 Department of Nephrology, The First Affiliated Hospital of Huzhou Teachers College, the First People’s Hospital of Huzhou, Huzhou, 313000 Zhejiang P.R. China; 2 Department of Pathology, The First Affiliated Hospital of Huzhou Teachers College, the First People’s Hospital of Huzhou, Huzhou, 313000 Zhejiang P.R. China

**Keywords:** Plasmablastic lymphoma, Proliferative glomerulonephritis with monoclonal IgG lambda deposits

## Abstract

**Introduction:**

As a very rare form of B-cell lymphoma, plasmablastic lymphoma (PBL) typically occurs in patients with underlying immunosuppression, including human immunodeficiency virus (HIV), organ transplantation, and autoimmune diseases. For HIV-positive patients, PBL normally originates in the gastrointestinal tract, especially from the oral cavity in most cases. It is extremely rare to find abdominal cavity involvement in PBL, and there has been no previously reported instance of proliferative glomerulonephritis with monoclonal immunoglobulin deposits (PGNMID) attributed to monoclonal IgG (MIgG) lambda secreted by PBL.

**Case presentation:**

We report the case of an HIV-negative female with nephrotic syndrome, renal insufficiency, and multiple swollen lymph nodes. Ascitic fluid cytology revealed a high level of plasmablast-like lymphocytes with the restriction of lambda light chains. Besides, the renal biopsy revealed PGNMID, which could presumably be secondary to MIgG-lambda-secreting by PBL. MIgG-lambda-restricted expression was discovered earlier in the kidney tissue than in the blood.

**Conclusion:**

The diagnostic landscape for PBL is notoriously intricate, necessitating a multifaceted and nuanced approach to mitigate the risks of erroneous identification.

## Background

Plasmablastic lymphoma (PBL), first described in 1997, [1] is an aggressive B-cell lymphoma that accounts for below 3% of all non-Hodgkin lymphomas. [2] In 2016, PBL was classified by the World Health Organization (WHO) as human immunodeficiency syndrome-associated lymphoma, which was considered to be related to infections with human immunodeficiency virus (HIV), Epstein-Barr virus (EBV), or other immunodeficiency states. [3] It has been mostly reported to originate from the oral cavity of patients with HIV infection [4] and in post-transplantation patients. [5–7] In some case studies on PBL in HIV-negative patients, it was reported that PBL occurred in other organs, for example, the testicles, [8] retroperitoneum, [9] and gastrointestinal tract. [10] However, PBL involving the kidneys is rarely reported. [11, 12]

Kidney injury could be induced directly by the malignancy, including ureteric obstruction [13] or neoplastic infiltration [14–16] of the renal parenchyma. The indirect influences of lymphoma, including hypercalcemia, [17] a hypercoagulable state, [18] renal vein thrombosis, [19] paraproteinemia, [20] immune-mediated glomerulonephritis (GN), [21] light chain deposition disease, ^[12 ]^ and amyloidosis, [22] also damage the kidneys. In clinical practice, renal failure is often caused by a combination of these factors. PBL is highly malignant and aggressive and has a poor prognosis. There are currently no effective treatments for this condition. Therefore, timely diagnosis and treatment are essential to prevent kidney damage.

Herein, we report a patient who presented with nephrotic syndrome and renal insufficiency due to proliferative glomerulonephritis with monoclonal immunoglobulin deposits (PGNMID) associated with PBL of the abdominal cavity. The case study in this work contributes a clinical report for the first time on PGNMID in PBL.

## Case presentation

A 77-year-old female patient initially presented with a clinical picture marked by generalized edema, anorexia, and a diminished urinary output spanning a three-month duration. Her medical history was significant for a decade-long battle with hypertension and a two-year history of jejunal and duodenal ulcers, compounded by non-atrophic gastritis. Histopathological analysis of biopsies obtained via gastrointestinal endoscopy disclosed abnormal lymphoid tissue hyperplasia. Physical examination was remarkable for a blood pressure reading of 167/75mmHg and pronounced 3 + lower extremity edema. Baseline serum creatinine levels, recorded two years prior to the current presentation, were noted to be 98µmol/L. Subsequent initial laboratory evaluations demonstrated a serum creatinine level of 117.3µmol/L, a hemoglobin concentration of 6.8 g/dL, an albumin level of 2.53 g/dL, and a 24-hour total protein level measuring 4577 mg. Serological screenings for viral infections, including HIV, hepatitis B, and hepatitis C, yielded negative results. Moreover, the autoimmune panel, which included anti-neutrophil cytoplasmic antibody (ANCA), antinuclear antibody, anti-DNA antibody, anti-glomerular basement membrane (GBM), and anti-phospholipase A2 (PLA2R) antibodies, was universally negative. Complement levels were within the normal range, as were the Immunoglobulin (Ig) A level of 5.22 g/L and the IgM level of 0.29 g/L; IgG levels were also normal. Bone marrow biopsy depicted hypercellular marrow characterized by erythroid and granulocyte hyperplasia, without any signs suggestive of lymphomatous infiltration. No monoclonal proteins were discerned via serum or urine protein electrophoresis. Tests for urinary Bence-Jones protein were negative, and serum free light-chain levels were within normative limits. Diagnostic imaging through ultrasound effectively ruled out urinary tract obstruction and renal vein thrombosis. Despite these comprehensive evaluations, the patient declined a renal biopsy and opted for symptomatic treatments, including antihypertensive agents, diuretics, and iron supplementation.

The patient was readmitted to the hospital 3 months later because of generalized edema, decreased urine output, dyspnoea after activities, and a slight cough. Physical examination revealed a high blood pressure of 172/82 mmHg and 2 + pitting edema of the lower extremities. The corresponding laboratory tests revealed renal insufficiency (creatinine: 185.3µmol/L), moderate anemia (hemoglobin: 8.4 g/dL), severe proteinuria (2989 mg/24 h), hypoalbuminemia(albumin:2.07 g/dL), hyperlipidemia (total cholesterol: 5.43 mmol/L; triglycerides: 1.38 mmol/L) and dysimmunoglobulinemia (IgA: 5.45 g/L; IgM:0.31 g/L) (Table 1).

**Table 1:** Laboratory data at admission

Parameters, unit	First medical evaluation	3 months later	9 months later		Normal reference
Blood					
White blood cell, ×10^3^/µL	6.0	6.5	6.2		3.5~9.5
Hemoglobin, g/dL	6.8	8.4	7.5		11.5~15.0
Platelet, ×10^3^/µL	245	229	207		125~350
Creatinine, µmol/L	117.3	185.3	136		33.0~98.1
Blood nitrogen, mg/dL	10.60	13.0	15.46		1.5~8.2
Sodium, meq/dL	144.9	145.4	141.8		137.0~147.0
Potassium, meq/dL	4.02	3.29	4.38		3.5~5.3
Total protein, g/dL	52.8	47.0	47.1		65.0~85.0
Albumin, g/dL	2.53	2.07	2.45		4.0~5.5
Total cholesterol, mmol/L	4.22	5.43	3.89		3.1~5.95
Triglyceride, mmol/L	1.02	1.38	1.50		0.56~1.69
Immunoglobulin A, g/L	5.22	5.45	9.60		0.7~5.0
Immunoglobulin G, g/L	7.2	5.9	1.89		7.0~16.0
Immunoglobulin M, g/L	0.29	0.31	0.23		0.4~2.8
Erythrocyte sedimentation rate,mm/h 92		69	14		0~20
Complement 3, g/L	1.2	1.13	0.944		0.9~1.8
Complement 4, g/L	0.35	0.35	0.296		0.1~0.4
Urine					
Occult blood	1+	3+	1+		Negative
24 h total protein, mg	4577	2989			20~150
Serum protein electrophoresis	No monoclonal protein	No monoclonal protein		Monoclonal protein	No monoclonal protein
Free light chains in serum κ free light chain λ free light chains κ : λ free light chain ratio	46 65 0.70	35 58 0.60		58 1665 0.03	6.77~22.4 mg/L 8.3~27.0 mg/L 0.26~1.65
Urine immunofixation	No monoclonal protein	No monoclonal protein		No monoclonal protein	negative

Renal sonography revealed bilaterally reduced kidney sizes (right and left kidneys of 9.4 and 9.8 cm, respectively) with increased echogenicity. Urine Bence-Jones protein test results, serum and urine immunofixation electrophoreses, and serum cryoglobulin levels were negative. Enlarged lymph nodes (bilateral cervical, axillary, supraclavicular, and inguinal) were detected. Histopathological examination of a biopsy of a supraclavicular lymph node showed lymphoid tissue hyperplasia. A second inguinal lymph node biopsy showed reactive hyperplasia with focal lymphatic sinus vascular transformation. After the patient received supportive treatment, the diuretic effect was poor, and the edema was obvious. Intravenous methylprednisolone 40 mg daily was administered before the renal biopsy, taking into the patient’s manifest gastrointestinal edema and their ability to rapidly attain therapeutic blood concentrations.

**Fig. 1:**
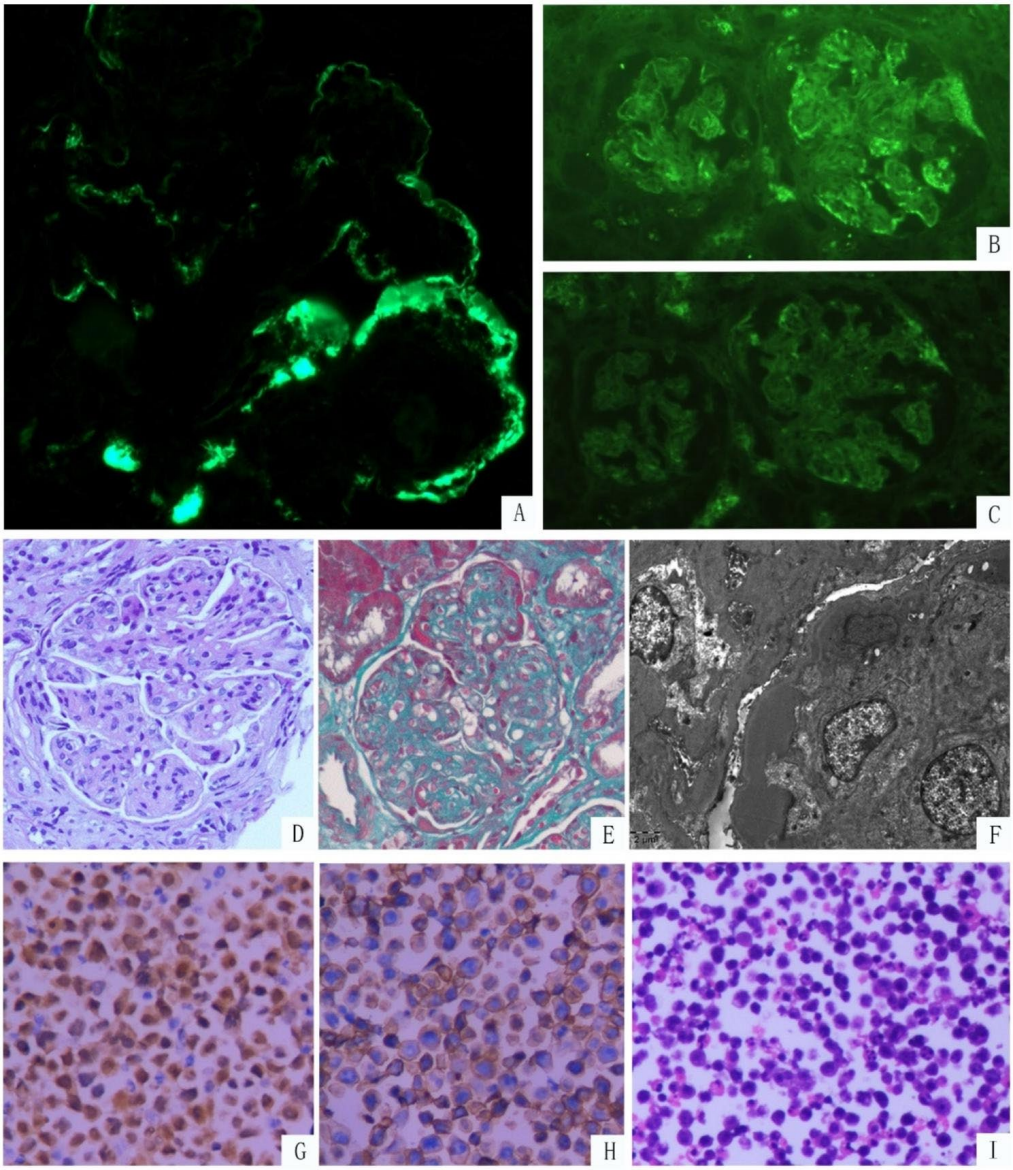
Renal biopsy and ascitic fluid findings (**A–C**) On IF, coarse, granular, predominantly capillary wall deposits are noted during IgG 3(2+) (Magnification, × 400) and lambda (2+) staining while negative for kappa staining (Magnification, both×100). (**D**) The membranoproliferative pattern of glomerular injury with lobular accentuation, mesangial matrix expansion, and hypercellularity, and occasional acellular mesangial nodule (Periodic acid–Schiff, magnification, ×400). (**E**) Broad and elongated subendothelial immune complex deposits and collagen fibers in the mesangium are observed (Masson’s Trichrome Staining, magnification, ×400). (**F**) Transmission electron microscopy image shows numerous subendothelial electron-dense deposits (Magnification, ×5000). (**G**) Immunohistochemical analysis of the ascitic fluid shows tumor cells with a strong expression of MUM-1(Magnification, ×100). (**H**) The tumor cells are strongly immunopositive for CD138 (Magnification, × 200). (**I**) Hematoxylin and eosin staining of the ascitic fluid reveals abundant plasmablast-like lymphocytes, presenting as round or oval-shaped cells with eccentrically located nuclei and distinct single or multiple nucleoli (Magnification, × 100)

A percutaneous renal biopsy was performed, and the histopathology is shown in Fig. 1. Light microscopy revealed 26 glomeruli, seven of which showed global sclerosis. The remaining glomeruli showed enlargement and hypercellularity, and the glomeruli showed accentuation of the lobular architecture, which expanded with the increase in the matrix and the cellularity. Segmental endocapillary proliferation of the mononuclear cells in the glomeruli was also observed. The glomerular capillary lumens were occluded, and the capillary walls were thickened, with double contours observed in many loops. No crescent lesions were observed, and protein reabsorption granules were detected in the tubules. Significant interstitial inflammation, severe tubular atrophy (60%), as well as interstitial fibrosis, were observed. Moderate intimal sclerosis of the arteries was also found.

Immunofluorescence (IF) studies revealed predominant IgG (2+) and C3 (+) staining in the capillary loops and mesangium. Lambda staining (2+) was stronger than kappa staining (-) in the capillary loops and mesangium. Staining results for IgA, IgM, C4, and C1q were negative. IF analyses of the tissues embedded in paraffin after pronase digestion were used to identify the IgG subtype, which revealed predominant IgG3 (2+) staining in the capillary loops; however, IgG1, IgG2, and IgG4 stainings were negative. Extraglomerular staining results were negative.

On electron microscopy (EM), the glomeruli showed diffuse, high-density, unorganized immune deposits located mainly in the mesangium and subendothelium, accompanied by segmental thickening of the glomerular basement membrane, mesangial insertion, and moderate podocyte foot process effacement. No substructures were identified. Congo red staining results were negative.

Immunohistochemical staining of the kidneys for Human Herpes Virus (HHV8) was negative. The final diagnosis was PGNMID. However, the patient refused treatment with cyclophosphamide, rituximab, or other biological inhibitors. She was continued on treatment with intravenous methylprednisolone 40 mg daily. The dose of methylprednisolone was tapered gradually and changed to oral methylprednisolone after half a month. Other treatments included anti-hypertensives, diuretics, erythropoietin-stimulating agents (ESAs), and iron supplementation. The patient’s blood results 20 days later showed a serum creatinine level of 136 µmol/L, a serum albumin level of 2.26 g/dL, and a hemoglobin level of 7.3 g/dL.

She was admitted 9 months later because of abdominal distension. An abdominal B-ultrasound showed a large effusion; the width of the upper abdominal cavity was approximately 75 mm, the width of the lower abdominal cavity was approximately 85 mm, the serum albumin level was 2.45 g/dl, the serum creatinine level was 136 µmol/L, and the hemoglobin level was 7.5 g/dl. A monoclonal IgG/lambda spike was detected on serum immunofixation electrophoresis. However, the urine immunofixation electrophoresis results were negative. She underwent a paracentesis. Cytology of the ascitic cell wax block exhibited a large number of plasmablast-like lymphocytes that were diffusely positive for CD38 and MUM-1 while negative for CD138, CD20, and CD3. Cell proliferation marker Ki-67 reached 80%. Additional immunostaining indicated that the PBL cells were lambda-light-chain-restricted. They were positive for IgG and CD56. The cells were negative for kappa light chains.

Immunophenotyping of lymphohematopoietic system tumors in the ascitic fluid indicated monoclonal hyperplasia of plasma cells, and plasma cell tumors were considered. HHV8 immunohistochemical staining of the ascitic fluid was negative. Based on the morphological and immunohistochemical findings, a final diagnosis of PBL was established. However, she refused CHOP (cyclophosphamide, vincristine, doxorubicin, and prednisolone) and CHOP-like treatments. Due to her poor economic circumstances, she also refused bortezomib and rituximab; therefore, she was continued on oral methylprednisolone 8 mg daily, thalidomide 50 mg nightly, and other supportive treatments such as anti-hypertensives, diuretics, calcium, amino acid, ESAs and iron supplementation.

. The anasarca did not improve significantly, and the patient strongly demanded discharge from the hospital. Unfortunately, she died at home soon after.

## Discussion

Although PBL rarely occurs, it is considered a highly aggressive form of B-cell lymphoma with an unknown pathogenesis that has a similar histomorphology to diffuse large B-cell lymphoma (DLBCL). PBL has been recognized as an independent subtype of large B-cell lymphomas and has been linked to HIV and EBV infections in the 2016 WHO classification of tumors of the lymphoid hematopoietic system. [3] The most commonly affected sites are the oral cavity and gastrointestinal tract, with kidney involvement being rare and mainly reported in HIV-positive patients. The incidence of HIV-negative PBL has been low; however, in recent years, the number of HIV-negative PBL patients has been increasing, especially in Asia, where there are very few HIV-positive patients. A statistical analysis of 60 patients with PBL in China showed that all were HIV-negative. [23] A previous study on 114 HIV-negative patients showed ubiquitous expression of plasma cell markers, including CD38, CD138, VS38c, and MUM1; EMA, CD45, and CD79a were positive to some degree, but CD20 was generally negative, and T cell markers, including CD3 and CD5, were also positive in some cases. [24] PBL normally contains EBV-encoded RNA (EBER1). [25] A light-chain restriction phenomenon is commonly found in PBL. [26] In this patient, 80% of Ki-67 staining showed positive immunohistochemical patterns for CD38, MUM-1, and CD56, and negative patterns for CD138 (-), CD20, and CD3. MIgG-lambda-restricted expression was observed in the kidneys, serum, and ascitic fluid in our patient.

The increase in the amount of serum-free lambda light chains raises the probability of lymphoma-induced kidney lesions. Few studies on kidney pathology in patients with PBL have been published, but a recent report showed that light chain cast nephropathy (LCCN) is related to bladder PBL. [12] However, studies on renal biopsy examinations of non-Hodgkin lymphoma patients have indicated multiple pathologies, such as minimal change disease, membranous nephropathy, membranoproliferative GN (MPGN), crescentic (rapidly progressive) GN, light chain proximal tubulopathy, cryoglobulinemic GN, amyloid light chain (AL) amyloidosis, immunotactoid GN, cast nephropathy, intracapillary monoclonal deposition, and intraglomerular large B-cell lymphomatous infiltration. [27, 28] The most common glomerular injury caused by lymphoma is MPGN, which is caused by abnormal clonal proliferation of B-cell lymphoma, leading to the production of autoantibodies, cryoglobulin or myeloma (M) protein secretion, and immune complex deposition in the kidney. [29].

It is widely known that a common and important reason for MPGN is a monoclonal gammopathy, [30] which correlates with single clone proliferation of plasma cells or Ig-producing lymphocytes, leading to the circulation of monoclonal IgGs (MIgGs). PGNMID is characterized by deposits typically composed of intact MIgGs with IgG heavy and light chains, of which IgG3-kappa is the most abundant. [31] Most PGNMIDs lack the symptomatology of hematologic diseases; although asymptomatic small plasma cell or small B cell clones may be present, they are classified as monoclonal gammopathies of renal significance (MGRS). [32] Most patients with PGNMID do not have a circulating monoclonal gammopathy as detected on serum and urine monoclonal protein testing, and their bone marrow aspirates and biopsies contain no plasma cell or B cell clones. Plasma cell clones are more likely to be detected on bone marrow biopsies if the MIgG is of the IgG1 subtype or if there are only light-chain deposits. [31].

Studies have shown that most glomerular diseases with immune complex deposition in patients with mantle cell lymphoma and most kidney pathologies are polyclonal, with only 2 cases of PGNMID. In one case, both the lymphomatous tissue and glomerular deposits contained monoclonal kappa, but no monoclonal kappa was detected in the blood. In another case, urine analysis showed IgG-lambda, but the kidney tissue showed IgG3-kappa type PGNMID, which differed significantly from the renal damage caused by plasma cells. [33] This highlights the importance of renal biopsy and considering lymphoma in the differential diagnosis when clonal inconsistency exists between the blood and kidney biopsy tissues.

In spite of supportive interventions, the patient continued to manifest severe edema and anorexia. Glucocorticoids are the cornerstone therapeutic agents for nephrotic syndrome. In light of the patient’s manifest gastrointestinal edema, which significantly compromised the potential for effective oral absorption, an alternative route for drug delivery was deemed essential. Intravenous steroids, due to their ability to rapidly attain therapeutic blood concentrations, emerged as the preferred approach. Consequently, intravenous methylprednisolone at a daily dose of 40 mg was initiated prior to a renal biopsy during her second hospital admission. The administration of methylprednisolone was gradually tapered and transitioned to oral methylprednisolone tablets. Post-treatment evaluations revealed a diminution of edema, an elevation in serum albumin levels from 2.07 g/dL to 2.45 g/dL, and a decrease in creatinine levels from 185.3µmol/L to 136µmol/L, thereby providing some evidence of therapeutic efficacy. However, it should be emphasized that glucocorticoids are not considered a standard treatment modality for PBL.

Prompt diagnosis and treatment to reduce or resolve monoclonal protein by eradicating the underlying clone are crucial for optimal renal recovery. [34] Standardized treatment for HIV-negative PBL has not yet been established, and experience with its treatment is mainly derived from case reports worldwide. Combination chemotherapy is often attempted as an initial treatment; CHOP and CHOP-like treatments are commonly used for PBL, with an overall response rate of approximately 70%. [35] Bortezomib, rituximab, and dexamethasone were administered to one patient with relapsed PBL, who eventually achieved near-complete remission. [36] Autologous stem cell transplantation (ASCT) has shown feasibility as a consolidation therapy for patients in remission after first-line therapy, especially those with high-risk factors. [37] Unfortunately, the patient refused chemotherapy and died. In summary, this was a case of senile nephrotic syndrome with elevated globulin levels, anemia, renal insufficiency, and generalized lymphadenopathy.

Our patient had a history of jejunal and duodenal ulcers, and the histopathological examination showed abnormal lymphoid tissue proliferation. Serum and urine immunofixation and bone marrow examination during the first two hospitalizations did not detect complete MIgG, but rather MIgG-lambda only in the renal tissue, leading to a diagnosis of PGNMID. MIgG lambda was detected using serum immunofixation electrophoresis during the third hospitalization. Bone marrow aspiration biopsy did not detect plasma cells or B cell clones, and no lymphomatous cells were found in the supraclavicular lymph node aspiration biopsy sample or inguinal lymph node surgical resection biopsy sample, which was consistent with the low probability of detecting monoclonal(M) protein in the blood, urine, or bone marrow in PGNMID. The cytology of the ascitic fluid indicated a large number of light-chain-restricted plasmablast-like lymphocytes. PBL, which is a non-Hodgkin’s lymphoma with a high degree of malignancy and invasiveness, was a possible diagnosis. The gastrointestinal tract was the most affected organ, visible through abnormal hyperplasia of the lymphoid tissue.

In conclusion, it is highly challenging to diagnose this type of lymphoma, and its manifestations are complex. If a lymphoproliferative disease and kidney damage are suspected, histopathological examination of the bone marrow, lymph nodes, serous cavity effusions, cerebrospinal fluid, spleen, and other organs is recommended to aid in the diagnosis.

## Data Availability

The data could be shared upon reasonable request to the corresponding author.

## Abbreviations

AL: amyloid light chain
ANA: antinuclear antibody
ANCA: antineutrophil cytoplasmic antibody
Anti-DNA: anti-double-stranded DNA
Anti-GBM: anti-glomerular basement membrane
ASCT: autologous hematopoietic stem cell transplantation
DLBCL: diffuse large B-cell lymphoma
EBER1: Epstein-Barr virus encoded RNA
EBV: Epstein-Barr virus
eGFR: estimated glomerular filtration rate
EM: electron microscopy
ESAs: erythropoietin-stimulating agents
GN: glomerulonephritis
HBV: hepatitis B virus
HCV: hepatitis C virus
HIV: human immunodeficiency virus
HHV8: human herpes virus
IF: immunofluorescence
LCCN: light chain cast nephropathy
MGRS: monoclonal gammopathy of renal significance
MIgG: monoclonal IgG
MPGN: membranoproliferative glomerulonephritis
M protein: monoclonal protein
PBL: plasmablastic lymphoma
PGNMID: proliferative glomerulonephritis with monoclonal immunoglobulin deposits
WHO: World Health Organization
